# The Prevalence of Skilled Birth Attendant Utilization and Its Correlates in North West Ethiopia

**DOI:** 10.1155/2015/436938

**Published:** 2015-10-04

**Authors:** Mulunesh Alemayehu, Wubegzier Mekonnen

**Affiliations:** ^1^Department of Public Health, College of Medicine and Health Sciences, Debre Markos University, P.O. Box 269, Debre Markos, Ethiopia; ^2^School of Public Health, College of Health Sciences, Addis Ababa University, Addis Ababa, Ethiopia

## Abstract

The low utilization of skilled birth attendants sustained high maternal mortality. The aim of this study was to assess its magnitude and correlates in Northwest Ethiopia. A study was conducted on 373 randomly selected women who gave birth in the 12 months preceding the survey. Correlates were identified using binary logistic regression. Skilled birth attendance was 18.8%. Inability to perform cultural practices in health facilities (65.5%), expecting smooth delivery (63.4%), and far distance (62%) were the main barriers. Women with urban residence (AOR = 5.46: 95% CI [2.21–13.49]), primary (AOR = 2.10: 95% CI [0.71–6.16]) and secondary-plus (AOR = 6.12: [1.39–26.92]) educational level, four-plus ANC visits (AOR = 17.33: 95% CI [4.22–71.29]), and proximity to health centers (AOR = 5.67: 95% CI [1.47–25.67]) had higher odds of using skilled birth attendants though women with no labor complications had lower odds (AOR = 0.02: 95% CI [0.01–0.05]). Skilled birth attendance use was low. Urban residence, primary-plus level of education, frequent ANC visits, living nearby the health centers, and a problem during labor were positively correlated with skilled birth attendance utilization. Stakeholders should enhance girls' education beyond primary level and ANC services and shorten distances to health facilities.

## 1. Background

Though pregnancy and childbirth are a natural phenomenon and is often an eventful process which most women aspire to have at some point in their lives, this normal life affirming process carries its own risk and complications to both the mother and the newborn [1, 2]. Globally over half a million reproductive age women (15–49 years) die every year from pregnancy and childbirth complications and 300 million women suffer from debilitating injuries [3, 4].

Many researchers documented that more than three-fourths of maternal deaths are related to direct obstetric causes, such as haemorrhage, sepsis, abortion, ruptured uterus, and hypertensive diseases of pregnancy which are easily preventable and treatable, and 77% of deaths occur during or soon after childbirth (within 24 hours) [3, 5]. Eighty-eight to ninety-eight percent of these problems are estimated to be avoidable, although over 99% of maternal deaths in Sub-Saharan Africa (SSA) could not be prevented [1, 2]. Maternal mortality in Ethiopia is the highest in the World with an estimated maternal mortality ratio of 676 deaths per 100,000 live births in 2011 which slightly increased from the 2005 maternal mortality ratio (MMR) level of 673 deaths per 100,000 live births [6, 7].

World leaders started to join their efforts together at the dawn of the millennium by the MDG through the safe motherhood initiative to decrease maternal morbidity and mortality globally [8]. Targets were set at the International Conference on Population and Development +5 (ICPD+5) to have more than 80% of deliveries assisted by skilled birth attendants globally by 2005, 85% by 2010, and 90% by 2015 [5]. In spite of all national and global efforts, the maternal and newborn morbidity or mortality indices have shown no change or only marginal reductions in the last five years indicating that MDGs targets by 2015 are unachievable using only current strategies [9]. One of the reasons for poor health outcomes among women and newborn is not using modern health care services by sizable proportion of women [10–12].

Studies from both industrialized and developing countries indicated that maternal mortality has been generally low when a higher proportion of deliveries are attended by skilled birth attendants [13–16]. However, study result from Ethiopia, Malawi, and Tanzania showed that socioeconomic status, availability of facility, short labor duration, staff attitudes, lack of privacy, reproductive behavior, cultural traditions, and the patterns of decision making power within household are mainly responsible factors for low utilization of skilled birth attendants [17–19]. According to WHO, skilled birth attendants are health professionals who have basic midwifery and obstetric skills including nurses, midwives, and physicians (WHO, 2004).

In Ethiopia, only 6% of births were delivered in health facilities at 2000 and there is no significant increase in the proportions of health facility delivery service utilization as evidenced from the series of Ethiopian Demographic and Health Surveys (EDHS) conducted in 2000, 2005, and 2011 which was found to be 6%, 8%, and 10% respectively [6, 7, 20].

Institutional delivery service utilization in Amhara Region was only 10.1% according to the EDHS 2011 which is equivalent to the national level of 10% [7]. However, according to the service statistics report by Akansha Guagusa Woreda Health Department, skilled delivery coverage was 21.7% in 2013 [21].

Therefore, this study was very crucial to measuring the prevalence of skilled birth attendance utilization and its correlates in remote areas of the second populous country in Africa to enhance maternal health to thereby reduce maternal morbidity and mortality in Ethiopia. The result will also be very important for policy makers as documented in the reproductive health road map to improve maternal health in general and increase level of skilled birth attendance by promoting quality antenatal care, make mothers have skilled assistance during their delivery, strengthen capacity of community health workers and community development army to perform emergency obstetric care (EmOC).

## 2. Methods

This study was conducted in Akansha Guagusa* Woreda* (district) in Awi zone, Amhara Regional State of Ethiopia. The main town of the study district was located at a distance of 450 kilometers northwest of Addis Ababa. The* Woreda *was administratively divided into seven clusters, 29 rural and 4 urban* kebeles* (the lowest administrative units). Clusters were established according to availability of health center. The area coverage of the study is 79881.75 square kilometers and was inhabited by about 221,796 persons [22].

According to the* Woreda* Health Department report there were seven health centres and five private clinics. With regard to health professionals employed in the* woreda*, there were nine health officers, five B. Sc. nurses, 46 clinical nurses, 16 midwifery nurses, one pharmacy technologist, nine pharmacy technicians, two laboratory technologists, 11 laboratory technicians, and 80 health service extension workers (primary health workers who had 10 months training on mainly disease prevention and promotion but do not have obstetric skills) making a total of 179 health professionals. The same source indicated that 28.4% mothers had four or more ANC visits and 74% of children had taken Penta three immunization. The average number of expected deliveries per annum in the* Woreda* was 6520, that is, 2.9% of the total population [21].

A community based cross-sectional study was conducted in February 2014 among 373 women who had delivered in 12 months prior to the study.

The sample size was calculated using single population proportion formula considering the Amhara Region skilled birth attendance utilization level of 10.1% [5], a 95% confidence level, 4% margin of error, 1.5 design effects, and 10% nonresponse rate. First, Akansha Guagusa Woreda (district) was purposely selected from eight Woredas from Awi zone. Three clusters in the Woreda, from available seven, were selected randomly. Two kebeles from each of the selected three clusters were also drawn randomly which made it a total of six kebeles. In each cluster, there are five health posts and one health center which cater approximately 5000 and 25,000 households and people, respectively. The number of women who delivered in the past one year (about 6520 in size) was obtained from registries of Health Services Extension Workers (HSEWs) in selected kebeles. To select the estimated sample size of 373, we used a multistage sampling scheme. The sample size was then proportionally allocated (using probability proportionate to size technique) according to the size of women who delivered in the past one year in each selected kebele. Then, in each kebele, study mothers were randomly selected from the rosters of HSEWs. Finally, study participants were identified using HSEWs as field guides.

An interviewer-administered pretested and close ended questionnaire developed in the local language was used. Mothers were briefed about the purpose of the study and data were collected after a verbal informed consent. The data collection was done by 12 female nurses supervised by two senior supervisors with public health background. Data quality was assured through careful design of the questionnaire and training of the field staffs. Moreover, data were checked for completeness and consistency.

Data were entered in Epi Info and exported to SPSS for analysis. Bivariate and multivariate models using binary logistic regression were run to assess any relationship between independent variables and skilled birth attendant utilization. Crude and adjusted odds ratios were used to ascertain effect sizes for any association between the dependent and predictor variables while significance was determined using 95% confidence intervals. Independent variables found to be significant with *p* value less than 0.05 at the bivariate level were included in a multivariate logistic regression model to control for potential confounding variables.

Ethical approval was obtained from the Research Ethics Committee of the School of Public Health, Addis Ababa University, and approval letter was obtained from the* Woreda* Administration Council. The purpose of the study was explained to mothers and the survey was commenced after obtaining verbal consent. Confidentiality of information was maintained by omitting any personal identifier from the questionnaires.

## 3. Results

Altogether, 373 mothers participated in the survey. Of these, more than three-fourths, 282 (75.6%), of them were from rural areas and almost all, 367 (98.39%), of them were Orthodox Christians. The main ethnic group was Agew, 290 (77.75%), and nearly one-third, 115 (30.83%), of them were in the age range of 25–29 years with a mean age of 30.41 (±5.56) years. With regard to educational status of respondents', nearly two-thirds, 233 (62.47%), of them could not read and write whereas only 54 (14.48%) of them had attained secondary educational level and above. Majority, 347 (93.03%), of them were currently married. Among married, 19 (5.5%) of respondents' husband were polygamous. Three quarters, 278 (74.53%), of the mothers were housewives and nearly half, 186 (49.87%), of them had five to seven household members. Concerning respondents' monthly income, majority, 124 (33.24%), of mothers were found in 701–1249 Ethiopian birrs income group which is less than two dollars per day (a dollar is approximately 20 ETB during our study period) (Table 1).

**Figure 1:**
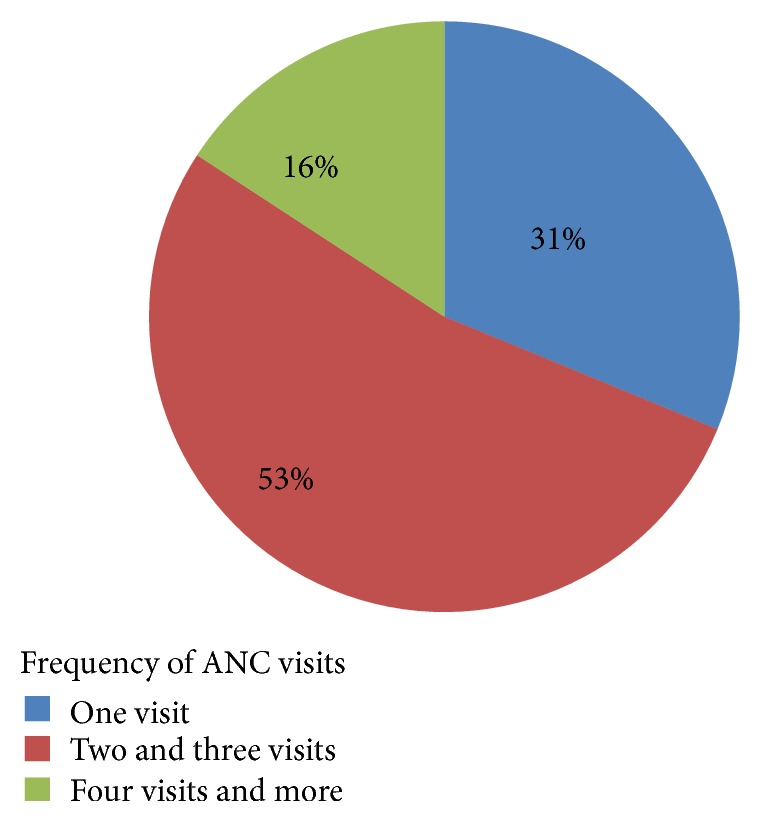
The distribution of respondents by their category of frequency of antenatal care visits in Akansha Guagusa* Woreda*, Awi zone, Amhara Region, Northwest Ethiopia, 2014.

Analysis of the obstetric characteristics of study women showed that 165 (44.24%) of respondents were married before celebrating their 16th birthday and the mean age at first marriage was 16.38 (±3.77) years. More than a third, 138 (37.00%), got their first pregnancy before the age of 19 years with the mean age of 19.73 (±3.00). About 101 (27.08%) gave their first birth before celebrating their 19th year of age with the mean age at first childbirth of 20.52 (±2.99) years. Moreover, 145 (38.87%) of them had more than four children and 285 (76.41%) of them who recently gave birth to a child had received antenatal care visits. Among 285 mothers who had ANC visit, about 89 (31%) of them had only one visit while 45 (16%) had four and more antenatal visits (Figure 1). Out of the 285 pregnant mothers, only 150 (52.63%) of them started ANC follow-up during the fourth month of their recent pregnancy.

In addition, this study revealed that only 78 (20.91%) of the mothers gave birth to their last babies at health facilities including health posts where health extension workers (who do not have education and training about basic obstetric skills) are working, whereas only 70 (18.77%) of them were attended by skilled birth attendants with basic obstetric skills (with nursing and above level of training). Among institutional deliveries assisted by skilled birth attendants, 43 (61.43%) of them delivered by spontaneous vaginal deliveries while 21 (30.00%) and 6 (8.57%) of them were assisted to give birth using instruments and through caesarean section, respectively. On the other hand, 71 (19.0%) of women who gave birth 12 months preceding the survey had history of labor complications, mainly prolonged labor, vaginal bleeding, and severe headache.

**Figure 2:**
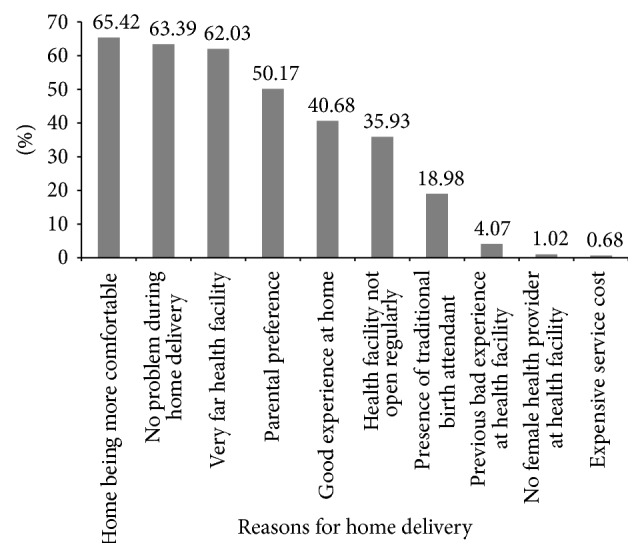
Reasons for home delivery among mothers in Akansha Guagusa* Woreda*, Awi zone, Amhara Region, Northwest Ethiopia, 2014 (*n* = 295).

Nevertheless, from mothers who were not attended by skilled birth attendants, 219 (58.71%) were assisted by family members and relatives, 60 (16.08%) by health extension workers, and 12 (3.22%) by traditional birth attendants, and 12 (3.22%) had to do it by themselves without any assistance. About 295 (79.09%) mothers gave a variety of reasons for delivering at home; of these, 193 (65.42%) felt more comfortable to deliver at home since they will perform variety of cultural practices, 187 (63.39%) assumed no problem during home delivery as it is natural, 183 (62.03%) considered that health facilities are located far from their places of residences, and for 148 (50.17%) of the mothers their parents decided where they should have a baby and preferred home as their place of delivery (Figure 2).

Binary logistic regression models were fitted to identify correlates of skilled birth attendant utilization. The first model which attempted to calibrate sociodemographic correlates revealed that residence type has been found to be strongly and significantly associated with skilled birth attendance at both bivariate and multivariate levels.

Skilled birth attendance was COR = 7.15: 95% CI (4.07–12.28) times more likely to be utilized among urbanites compared with rural residents whereas when other confounding sociodemographic variables were controlled, the odds of skilled birth attendance utilization were AOR = 5.46: 95% CI (2.21–13.49) times higher among urban residents compared with their rural counterparts. On the other hand, skilled birth attendance was COR = 2.59: 95% CI (1.33–5.05) and COR = 9.83: 95% CI (4.95–19.52) times more likely to be utilized among mothers who completed primary and secondary and above level of education, respectively, compared with those who had never been into formal schooling. However, the significance of association vanishes at multivariate level for primary level of education. When all the other sociodemographic variables are controlled, the odds of skilled birth attendance were AOR = 6.12: 95% CI (1.39, 26.92) times higher among those mothers whose educational level was secondary and above compared with those with no education (Table 2).

The focus of the second model was to identify the reproductive health related correlates of skilled birth attendance utilization. Skilled birth attendant utilization among women who had four or more antenatal care visits was AOR = 17.33: 95% CI (4.22–71.29) times higher compared to those mothers with three and lower antenatal care visits (Table 3). On the other hand, women who had been living in an area nearby the health center were more likely to be assisted by skilled birth attendants in their parturition time than those mothers living closer to health post (AOR = 5.67: 95% CI [1.47–25.67]) (Table 3). Moreover, the odds of skilled birth attendant utilization among mothers who did not encounter labour complication during current delivery was 98% times less likely to deliver with the assistance of skilled professionals than women who encountered complication (AOR = 0.02: 95% CI [0.01–0.05]) (Table 3).

## 4. Discussion

This study showed that about one-third of the women in Akansha Guagusa Woreda had completed three visits of antenatal care services. However, institutional delivery and skilled birth attendant utilization were low, especially among women from rural areas, who had three and less antenatal care visits and were uneducated.

In this study less than one-fifth of the mothers (18.77%) were assisted by skilled birth attendants during their recent deliveries. Moreover, the study documented women who were not attended by anyone during delivery. The prevalence of skilled birth attendant utilization for the study district was somewhat similar to the findings of other studies done in Ethiopia, that is, Ephrtanagidim district, Kembata-Tembaro zone, and Raya Alamata district [23–25]. However, it was higher than the 2011 Ethiopian Demographic and Health Survey finding, a study done in Sekela district, Metekel zone of Ethiopia, and Afghanistan [7, 14, 23, 26]. This might be due to improvements in accessing and utilizing the service and community mobilization through health developmental army which has been implemented in the country in recent years. On the other hand, it was lower than those studies conducted outside of Ethiopia (Southern Tanzania, Nigeria, Namibia, and Nepal) and in Ethiopia (Bahir Dar, Woldia, and Kilte Awlaelo) [8, 12, 26–30]. The differences could be explained by the fact that women in those countries had better economic status, educational status, and antenatal care service coverage. However, Ethiopian studies were done in urban contexts for which ANC coverage is higher.

This study also showed the association of place of residence with skilled birth attendant utilization. In particular, mothers who resided in urban areas were more likely to get skilled birth attendance compared to their counterparts which is consistent with the findings of similar studies done in Ethiopia and other countries [4, 8, 13, 14, 24, 25, 29–35]. This might be due to the increased availability and access to health services and other infrastructures such as shorter distance to health facilities, better roads and transportation, and more information and education in urban than rural areas. The media promoting good health have been widely available in urban areas and rural residents might be influenced by traditional practices.

Educational status of mothers was also significantly associated with the utilization of skilled birth attendants. Mothers who had attained primary and secondary and above educational level were more likely to utilize skilled birth attendant than those mothers who were unable to read and write. This finding was in line with other studies that were done in Ethiopia and other developing countries [8, 12–14, 25, 31–34, 36, 37]. This could be due to the fact that educated women might have more access to written information and could adapt to modern cultural perspectives. Moreover, education empowers women so as to increase autonomy and self-confidence to make them decide for their better reproductive health needs.

Controlling other variables, frequency of antenatal care visits during their last pregnancy was also found to be a strong predictor of utilization of skilled birth attendants. Those mothers who had four antenatal care visits and above were more likely to deliver through the assistance of skilled birth attendants than those mothers who had three and less antenatal care visits. This finding was consistent with studies done in developing countries including Arsi, Alamata, Tigray, Dabat, Woldia, Mekelle, Fogera in Ethiopia, Nepal, Tanzania, and Cambodia [4, 8, 13, 14, 24, 25, 29–35]. This might be due to the fact that as the number of antenatal care visits increases, women will be acquainted with basic information on pregnancy and delivery related risks that require skilled providers' assistance.

Another important finding of this study was that mothers who did not face any labour complications during recent labour and delivery were 98% less likely to get skilled attendant during their delivery compared to those mothers who faced maternal complications. This implies that labouring mothers decide to give birth in health facilities and require the assistance of skilled birth attendants when they faced difficulties and when repeated trials at home failed. Similar findings were documented by other studies done in Sekela, North Shewa, Agemssa, Wollega, and Metekel zone [13, 14, 23, 38, 39].

## 5. Conclusions

Utilization of skilled birth attendant is still very low with a high number of deliveries being attended by unqualified persons at home. There are still women who deliver by themselves. Women's higher education, urban residence type, higher frequency of antenatal care visits, proximity of living to a health center, and encountering complications during current labour were found to be positively correlated with skilled birth attendant utilization. Therefore, the government should enhance secondary and above level of educational attainment among females, promote universal antenatal care follow-up service, and encourage mothers to utilize skilled birth attendants during pregnancy and delivery. Good referral linkages should be established, health facilities should be staffed by professionals with basic obstetric skills and with necessary supplies and medicines, and different means of behavioral changing communication should be designed to improve the demand.

## Figures and Tables

**Table 1:** Sociodemographic characteristics of respondents in Ankasha Guagusa *Woreda*, Awi zone, Amhara Region, Northwest Ethiopia, 2014 (*n* = 373).

Characteristics of respondents	Numbers	Percent
Residence		
Rural	282	75.60
Urban	91	24.40
Religion		
Orthodox	367	98.39
Muslim	6	1.61
Ethnicity		
Agew	290	77.75
Amhara	83	22.25
Woman's education		
No education	233	62.47
Primary	86	23.06
Secondary and above	54	14.48
Age group		
≤24	53	14.21
25–29	115	30.83
30–34	110	29.49
≥35	95	25.47
Marital status		
In marital union	347	93.03
Not in union^†^	26	6.97
Polygamous		
No	328	94.5
Yes	19	5.5
Husband's education		
No education	192	55.33
Primary	88	25.36
Secondary and above	67	19.31
Woman's occupation		
Housewife	278	74.53
Others^††^	95	25.47
Husband's occupation		
Farmer	264	76.08
Others^††^	83	23.92
Household size		
≤4	130	34.85
5–7	186	49.87
≥8	57	15.28
Monthly income (Eth. Birr)^++^		
≤700	61	16.35
701–1249	124	33.24
1250–1799	121	32.44
≥1800	67	17.96
Source of income		
Women themselves	54	14.5
Family and relatives	52	13.9
Both women and husbands	178	47.7
Husbands	89	23.9

^†^Not in union: single, divorced, and widowed and ^††^others: employee (government and private), petty traders, daily laborer, student, and handcrafter.

^++^Monthly income is calculated by converting annual crop yields and other agricultural products they had in that year into monthly cash income using the then market price of each merchandise and ultimately dividing it by 12.

**Table 2:** Sociodemographic characteristics associated with skilled birth attendance in Akansha Guagusa *Woreda*, Awi zone, Amhara Region, North West Ethiopia, 2014.

Variables	SBA use		Crude odds ratio (95% CI)	Adjusted odds ratio (95% CI)
	No	Yes		
Residence				
Rural	253	29	1.00	1.00
Urban	50	41	**7.15 **(4.07–12.58)^**∗∗∗**^	**5.46 **(2.21–13.49)^**∗∗∗**^
Women's education				
No education	210	23	1.00	1.00
Primary	67	19	**2.59 **(1.33–5.05)^**∗∗**^	2.10 (0.71–6.16)
Secondary and above	26	28	**9.83 **(4.95–19.52)^**∗∗∗**^	**6.12 **(1.39–26.92)^**∗**^
Polygamy				
No	271	57	**0.36 **(0.14–0.96)^**∗**^	0.36 (0.12–1.16)
Yes	12	7	1.00	1.00
Husbands' education				
No education	172	20	1.00	1.00
Primary	77	11	1.23 (0.56–2.69)	0.68 (0.23–1.98)
Secondary and above	34	33	**8.35 **(4.29–16.25)^*∗∗∗*^	2.04 (0.45–9.33)
Women's occupation				
Housewife	245	33	1.00	1.00
Others^+^	58	37	**4.74 **(2.73–8.21)^**∗∗∗**^	0.07 (0.00–2.00)
Husbands' occupation				
Farmers	234	30	1.00	1.00
Others^†^	49	34	**5.41 **(3.03–9.66)^**∗∗∗**^	7.72 (0.17–83.54)
Household size				
2–4	94	36	**5.07 **(1.29–6.61)^**∗∗∗**^	0.83 (0.52–6.37)
5–7	156	30	1.06 (0.41–2.76)	0.76 (0.55–5.62)
8–12	53	4	1.00	1.00
Source of income				
Women themselves	36	18	**2.92 **(1.92–6.61)^**∗∗**^	1.43 (0.49–4.16)
Relatives and family	44	8	1.06 (0.41–2.76)	1.79 (0.58–5.49)
Both women and husbands	147	31	1.23 (0.61–2.49)	0.82 (0.34–1.99)
Husbands	76	13	1.00	1.00

Significance at ^*∗*^ *p* value < 0.05, ^*∗∗*^ *p* value < 0.01, and ^*∗∗∗*^ *p* value < 0.001.

^+^Others: governmental workers, housewives, merchants, and daily laborers for respondents.

^†^Others: governmental workers, merchants, and daily laborers for husbands.

**Table 3:** Obstetric characteristics associated with skilled birth attendance in Akansha Guagusa *Woreda*, Awi zone, North West Ethiopia, 2014.

Variables	SBA use		Unadjusted odds ratio (95% CI)	Adjusted odds ratio (95% CI)
	No	Yes		
Age at first marriage				
8–15 years	148	17	**0.21 **(0.11–0.40)^**∗∗∗**^	1.64 (0.35–7.64)
16–18 years	84	14	**0.30 **(0.15–0.60)^**∗∗**^	0.76 (0.25–2.28)
19–27 years	71	39	**1.00**	1.00
Age at first pregnancy				
14–18 years	120	18	**0.53 **(0.30–0.95)^**∗**^	0.44 (0.11–1.67)
19–31 years	183	52	**1.00**	1.00
Parity				
1–4 children	173	55	**2.76 **(1.49–5.09)^**∗∗**^	1.82 (0.68–4.88)
5–12 children	130	15	1.00	1.00
Frequency of ANC visit				
One visit	78	11	1.00	1.00
Two and three visits	121	30	1.76 (0.83–3.71)	2.52 (0.81–7.83)
Four visits and more	22	23	**7.41 **(3.14–17.52)^**∗∗∗**^	**17.33 **(4.22–71.29)^**∗∗∗**^
Type of nearby HF				
Health post	253	29	1.00	1.00
Health center	50	41	**7.15 **(4.07–12.58)^**∗∗∗**^	**5.67 **(1.47–25.67)^**∗**^
Travelling hour				
<1 hour	81	46	**9.94 **(2.29–43.23)^**∗∗**^	3.75 (0.54–25.67)
1 hour	187	22	2.06 (0.46–9.15)	1.50 (0.25–9.08)
>1 hour	35	2	1.00	1.00
Ever given birth at HF				
No	240	36	1.00	1.00
Yes	63	34	**3.60 **(2.09–6.20)^**∗∗∗**^	0.77 (0.29–2.04)
Problem during current labour				
No	283	22	**0.04 **(0.02–0.07)^**∗∗∗**^	**0.02 **(0.01–0.05)^**∗∗∗**^
Yes	23	48	1.00	**1.00**

Significance at ^*∗*^ *p* value < 0.05, ^*∗∗*^ *p* value < 0.01, and ^*∗∗∗*^ *p* value < 0.001.
